# Tomato Processing By-Products Valorisation through Ohmic Heating Approach

**DOI:** 10.3390/foods12040818

**Published:** 2023-02-15

**Authors:** Marta C. Coelho, Soudabeh Ghalamara, Débora Campos, Tânia Bragança Ribeiro, Ricardo Pereira, António S. Rodrigues, José A. Teixeira, Manuela Pintado

**Affiliations:** 1 Universidade Católica Portuguesa, CBQF—Centro de Biotecnologia e Química Fina—Laboratório Associado, Escola Superior de Biotecnologia, Rua Diogo Botelho 1327, 4169-005 Porto, Portugal; 2 CEB—Centre of Biological Engineering, University of Minho, 4710-057 Braga, Portugal; 3 Centre for Toxicogenomics and Human Health, Genetics, Oncology and Human Toxicology, NOVA Medical School, Faculdade de Ciências Médicas, Universidade Nova de Lisboa, Campo dos Mártires da Pátria 130, 1169-056 Lisbon, Portugal

**Keywords:** *Lycopersicum esculentum*, new technologies recovery, wastes, bioactive compounds, phenolic compounds, carotenoids

## Abstract

Tomato by-products from processing industries have a higher potential to be reused as a source of bioactive compounds. Reliable national data on tomato by-products and physicochemical characterisation that will inform and find effective planning on tomato waste management in Portugal is absent. To help obtain this knowledge, selected Portugal companies were recruited to obtain representative samples of by-products generation, and physicochemical composition was evaluated. Furthermore, an environmental-friendly method (the ohmic heating (OH) method, which allows the recovery of bioactive compounds in absence of hazardous reagents) was also used and compared with conventional methods to explore new safe value-added ingredients. Total antioxidant capacity and total and individual phenolic compounds were also evaluated by spectrophotometric and high-performance liquid chromatography (HPLC), respectively. Tomato processing by-products have revealed a higher potential since both collected samples from companies were rich in protein (between 16.3 to 19.4 g/100 g DW, with fibre content ranging from 57.8 to 59.0 g/100 g DW). In addition, these samples contain 17.0 g/100 g of fatty acids (mainly polyunsaturated, monounsaturated and saturated, such as linoleic, oleic, and palmitic acid, respectively). Also, they present mainly chlorogenic acid and rutin as phenolic compounds. After understanding its composition, the OH was applied to determine added-value solutions to tomato by-products. With extractions, two types of fractions were obtained, namely liquid fraction rich in phenols, free sugars, and carotenoids and a solid fraction rich in fibre bound to phenols and carotenoids. This treatment has been shown to have the ability to preserve carotenoids, such as lycopene relative to conventional methods. Nevertheless, new molecules were identified by LC-ESI-UHR-OqTOF-MS analysis, such as phene-di-hexane and N-acethyl-D-tryptophan. According to the results, the OH boosts the potential of tomato by-products and can be directly introduced into the process, contributing to the circular economy and zero by-products.

## 1. Introduction

An expanding global human population, rapid urban development, and economic growth are increasing waste production at an alarming rate. By 2050, the world is predicted to generate 3.40 billion tons of waste per year, a significant increase from today’s 2.01 billion tons [1,2]. Decoupling economic development from resource demands is essential given the ever-increasing demands on our planet’s limited resources. However, the shift to an eco-friendlier economy is a cause for concern and involves joint action by multiple stakeholders across borders. Stemming from an in-depth assessment of sustainable development goals on responsible expenditure and industry, in recent years a wide variety of stakeholders (political, scientists, financials, industry) have discussed and progressed on this shift to the circular economy as a means for accelerating the implementation of multiple sustainable development goals. It is vital that companies progressively adapt the strategies for more resource-efficient use. Traditionally, the system has been linear, where life, food, and other resources are utilized and then wasted. In a circular system, the idea is that materials will be repeatedly recycled in some way or another rather than ending at the landfill [1,3].

*Lycopersicon esculentum* L. (tomato) is one of the essential fruits consumed worldwide. According to the Food and Agriculture Organization of the United Nations [4], in 2018, the global tomato area harvested was about 5 million hectares, producing approximately 173 million tons (fresh and processed) with a total gross production value of 85 billion US dollars. This fruit is consumed in the form of processed products such as tomato juice, paste, puree, ketchup, and salsa. Interestingly, this agro-industry is known to produce significant amounts of solid by-products, namely seeds, peels, pulp and fibrous parts that account for 7.0–7.5% of raw materials [5]. These are rich in bioactive compounds (BC), such as phenolic compounds, carotenoids, lycopene, β-carotene, fibre, and protein, which are related with health benefits which includes anti-inflammatory and chemopreventive properties and cardiovascular protection [6]. The by-products generated are mainly destined for animal feed without any processing or dumped in landfills, thus representing costs and environmental concern for the tomato processing industry, which must be carefully considered [6,7]. Fortunately, food industries and researchers have started to pay special attention to this by-product to recover their bioactive compounds (e.g., phenols, carotenoids, vitamin E, and sterols for applications in food pharmaceutical and or/cosmetic industries).

To accomplish the objectives implied in the concept of circular economy and biorefinery, new approaches with green technologies to obtain different fractions with zero-waste must be evaluated. 

Traditionally, to isolate and recover bioactive compounds, such as phenols and carotenoids from tomato by-products, liquid-liquid extraction has been employed. This method uses a large amount of hazardous organic solvents that adversely affect both health and the environment. Furthermore, it may jeopardize its reuse, such as the possibility to incorporate into foods [8] and therefore to be maintained in the food value chain.

In ohmic heating (OH) an electric current is carried directly through materials with electrical resistance, creating heat and raising the product’s immediate and uniform temperature. It has become a promising technology to be applied to food products or by-products with some advantages, including preserving nutritional, functional, and structural properties of products [9,10,11]. This method is also fast, and homogeneous with efficient energy transfer technology providing an environmentally clean methodology, reducing processing costs, and improving the products added value. This technology was used by [12] to analyse the effects of voltage gradients on tomato paste, obtaining electrical conductivity data and verifying the possibility of OH application in tomato industries. These authors observed significant differences in heating time and pH changes of samples caused by voltage gradient application, opening the possibility to the usage of this technology at industrial levels to obtain processed tomato with preserved properties. Reduced process times in OH maintains the nutritional and sensory properties of nutrients. OH inactivates positively antinutritional elements such as lipoxygenase (LOX), polyphenol oxidase (PPO), and pectinase, removing their dynamic metal radicals by electric field application [13,14]. Furthermore, this technique has been applied for several purposes, such as dairy products pasteurisation and processing juices. However, few studies have reported the application of OH on food by-products. Studies reported the possible use of this technology in tomato paste by-products and wine by-products to recover bioactive compounds. Also, it is possible to obtain carotenoids or phenolic compounds by changing OH conditions such as temperature and time of reaction. Additionally, the effects of OH on bioactive compounds and in particular their recovery from by-products are still scarce. Other disadvantages include the required modification based on the conductivity of the dairy liquid, which is difficult to monitor and manage; and the intricate coupling between temperature and electrical field distribution. Thus, the purpose of this work was to assess the phytochemicals profile of tomato by-products (phenols and carotenoids) and test for the first time the application of OH as an alternative to traditional liquid-liquid extraction to valorise this by-product through the extraction of bioactive compounds. 

## 2. Materials and Methods

### 2.1. Materials

The 2, 20-azo-bis-(2-methylpropionamidine)-dihydrochloride (AAPH), fluorescein, 2, 2-azinobis-3-ethylbenzothiazoline-6-sulphonic acid (ABTS diammonium salt), potassium sorbate, sodium carbonate, and ethylenediaminetetraacetic acid (EDTA were purchased from Sigma-Aldrich (Sintra, Portugal). Hexane, ethanol, Folin–Ciocalteu’s reagent, and potassium persulfate were purchased from Merck (Algés, Portugal). Standards of ascorbic acid, Trolox, gallic acid, rutin, p-coumaric, and 4-hydroxybenzoic acid, were purchased from Sigma-Aldrich (Sintra, Portugal), while kaempferol, β-carotene, lycopene, zeaxanthin, and lutein (Extrasynthese, France) were purchased from Extrasynthese (Lyon, France).

### 2.2. Samples Preparation

Tomato processing by-products were obtained from two industrial processing companies in Portugal, Italagro from Vila Franca de Xira and Sugal Group from Benavente. Both organizations used tomatoes of Heinz cultivar to produce different processed tomato products. The tomato from the first company was obtained fresh (C1), right after the stage in which the skins and seeds of the tomato were removed while processing the pulp; for the other sample, it was delivered to us after being dried in the sun (C2), to remove excess of humidity.

#### Samples Processing and Fractions Production

The by-products formed during the tomato processing were immediately collected, stored at vacuum bags and transported at 4 °C in a portable refrigerator. The samples received were analysed in terms of phytochemical composition, dry matter, ashes, proteins, fibre, sugars, and fatty acids. After phytochemical composition both samples were mixed, forming a blend. The blended sample was fractionated in four types of processing: (i) received sample; (ii) drying at 55 °C in a convection oven for 24 h; (iii) frozen at −20 °C for 24 h; and (iv) frozen at −80 °C in an ultra-low temperature freezer for 24 h to analyse the impact of these processes on tomato by-products. After the different processing, the samples were all dried (50.0 °C ± 3.0, during 24 h), milled with a food processor (Bimby, Vorwerk, TM5, Wuppertal, Germany) and sieved with a mechanical sifter using sieves of pore sizes 150 μm (particle size 150 µm) in the same conditions [15,16]. 

Finally, the samples kept at −20 °C were submitted to conventional and ohmic heating extraction methods, applying ethanol as a food-grade solvent.

### 2.3. Extraction Methodologies Employed

#### 2.3.1. Phenols Conventional Extraction

Phenol extraction was performed according to [16] with slight modifications. Tomato bagasse (2.5 g) was homogenised using an ultra-turrax (IKA, T18, Wilmington, NC, USA) for 2 min with 25 mL of methanol (80%, *v*/*v*) at room temperature., followed by 30 min (300 rpm) stirred and centrifuged at 8000× *g*, at 4 °C, during 5 min. The resulting supernatant (liquid fraction) was analysed in terms of antioxidant activity, phenolics content and phenolics quantitative profile. The solid fraction was analysed for bound phenolic compounds to fibre. Both extracts, liquid and solid fraction were stored at −80 °C.

#### 2.3.2. Carotenoid Conventional Extraction 

A carotenoids conventional extraction method was conducted to compare and characterise the carotenoids compounds present in the by-products. Briefly, 2.5 g of tomato by-products were suspended in 5 mL of cold ethanol absolute and homogenised at 14,000× *g* for 3 min applying an ultra-turrax. Hexane ≥ 99% (4 mL) was added to the homogenate, and the resulting mixture was homogenised for an additional 2 min and then centrifuged for 10 min at 4000× *g*. The hexane layer containing the carotenoids was transferred to a polypropylene tube. A solution of saturated sodium chloride (2.5 mL) and an additional 4 mL of hexane were added to the slurry, and the resulting mixture was homogenised for 1 min. The mixture was centrifuged as described in the previous step, and the second hexane layer recovered and mixed with the first hexane layer [17]. The extracts (liquid fraction) and retentates (solid fraction) were stored at −80 °C. The liquid extract was analysed for total carotenoids content and a carotenoid profile by spectrophotometric and HPLC UV/VIS DAD. All extracts and analyses were performed in triplicate.

#### 2.3.3. Ohmic Heating Extraction

An OH system was used to extract bioactive compounds from tomato by-products to compare with a conventional method. The OH method, very briefly it consists of a cylindrical glass reactor (30 cm total length with 207 cm diameter) with two stainless steel electrodes placed at each edge isolated by polytetrafluoroethylene (PTFE) caps. The bioactive extraction was performed based on previous studies [18]. A hydroethanolic solution (ethanol 30%, *v*/*v*) was used, for 15 min at 55 °C, 25 kHz of frequency and a supplied voltage of 60 v. The extracts were centrifuged and both solid and liquid fractions were stored at −80 °C. The liquid extracts were analysed in terms of total phenols content, total carotenoids and total antioxidant capacity, also the carotenoids and phenols profiles were evaluated by HPLC. On the other hand, the solid by-products were analysed in terms of fibre and bound phenols profile. All analyses were performed in triplicate.

### 2.4. Compositional Analysis

#### 2.4.1. Proximate Composition

The moisture content of fresh tomato bagasse (including seeds, peels, rest of pulp and fibrous parts) was determined according to the oven method [19,20]. 

The ashes (inorganic deposit remaining after the water and organic matter have been discarded by warming with oxidising agents, which gives a proportion of the aggregate sum of minerals inside a nourishment) were determined according to the AOAC official method 942.05. Briefly, one gram of tomato bagasse was weighed (w_1_) into a previously weighed crucible (W_0_) and placed in the muffle furnace at 600 °C for 2 h. After cooling, it was placed in a desiccator and the crucible weighed at room temperature (w_2_). 

Total nitrogen was analysed by the Kjeldahl method, and protein content was then calculated using a conversion factor of 6.25. 

A fat extraction from tomato by-products was performed by hydrolysis with ether. The fat content was determined gravimetrically by the Soxhlet method using petroleum ether (boiling point 60–80 °C), according to the method described in [21]. Subsequently, it was methylated to obtain fatty acid methyl esters. The individual fatty acids characterisation and quantification was analysed by gas chromatography (GC-FID). All analyses were performed in triplicate.

#### 2.4.2. Fibber

The crude fibre content was determined with an acid/alkaline hydrolysis of insoluble by-products. All methodologies followed the recommendations of the Official Methods of Analysis [21,22]. All measurements were carried out in triplicate. The chemical compounds were expressed as g/100 g dry weight (DW) and all analyses were performed in triplicate.

#### 2.4.3. Cellulose, Hemicellulose and Lignin

Both carbohydrates and lignin were accurately measured in all flours (tomato bagasse, solid residues obtained after ohmic and conventional extractions), according to [21,22]. The method employed two-step sequential acid hydrolysis, 72% sulfuric compound at the beginning followed by 4% (*v*/*v*) sulfuric compound at the second measure to hydrolyse both cellulose and hemicellulose to sugars and quantified via HPLC. So, the carbohydrates (sugars) issued were assessed to determine the hemicellulose sugar content. The results were expressed in g/100 g DW and all analyses were performed in triplicate.

#### 2.4.4. Total Pectin

The total pectin content of fresh samples was calculated based on the method described by [23], comprising the three fractionated pectin water-soluble pectin (WSP), chelator soluble pectin (CSP) and hydroxide soluble pectin (HSP). All analyses were performed in triplicate. The results were expressed in galacturonic acid equivalents (GUAE)/100 g.

### 2.5. Total Antioxidant Capacity

The antioxidant activity was performed using ABTS and ORAC methods. The ABTS was performed according to [24] with slight modifications. The sample was added to a coloured solution of 2,2′-azinobis-(3-ethylbenzothiazoline-6-sulfonic acid radical cation) (ABTS^•+^), with an optical density (OD) measured at 734 nm and adjusted to 700 ± 0.020 in a spectrophotometric microplate reader (Sunrise Tecan, Grödig, Austria). After 6 min of reaction, the final OD was read and the results were given in ascorbic acid equivalent. 

The ORAC measurement of the different extracts of tomato by-products was assessed according to [25]. The extracts were dissolved with phosphate buffer (pH 7.4), and the Trolox standard curve (0–90 mg/L) was performed. At the time of analysis, 70 nM fluorescein and 14 mM AAPH results were made at ORAC buffer. The 96 wells coloured microplate was prepared to contain25 µL of blank control (ORAC buffer); standardised, control, or sample and 200 µL of fluorescein solution were added [25]. After, 50 µL of newly prepared AAPH solution was added. The microplate was incubated for 10 min at 37 °C. The fluorescence readings were carried every 2 min within 104 cycles using the FLUO star OPTIMA plate reader (BMG Labtech, Offenburg, Germany). The wavelength excitation was 485 nm, and the emission was 530 nm. Results were expressed in µmol Trolox equivalent/g DW and the measurements of each sample were performed in triplicate.

### 2.6. Total Phenols Content and Free and Bound Phenolic Compounds

The Total Phenols Content (TPC) were evaluated through Folin–Ciocalteu spectrophotometric method. A mixture of a sample (5 µL), Folin–Ciocalteu reagent (15 µL), sodium carbonate at 75 g/L (60 μL), and distilled water (200 μL) was performed. Next, samples were heated at 60 °C for 5 min, and the OD was read at 700 nm using a spectrophotometric microplate reader (Sunrise Tecan, Grödig, Austria). TPC was expressed as a milligram of gallic acid equivalent per dry weight material (mg GAE/g). The analyses were performed in triplicate, and a standard deviation was calculated. 

The bound phenolic analyses were performed to the tomato bagasse samples, as well as to the solid residues obtained after conventional and ohmic extractions. The tomato bagasse was washed three times with ethanol absolute to eliminate the free phenols. Concerning the other samples obtained after extraction, they were already washed during the extraction process. Then, for all of the samples, a reaction of 4 h was carried out with 20 mL of NaOH (4 M) and 1 g of washed residue. The solution obtained was acidified with HCl (6 M) at pH 1.5 to 2.0 and then centrifuged for 30 min at 12,000× *g* The extraction was then performed with ethyl-acetate, for 15 min, five times [26]. The supernatant was concentrated in a vacuum evaporator, resuspended with 10 mL of ethanol absolute. The extracts obtained of the free total content of phenols and the bound phenols were analysed by HPLC. 

### 2.7. Determination of Total Carotenoids, Lycopene, β-carotene and Chlorophylls Content

Total Carotenoids (TC) content of tomato bagasse were assayed using a spectrophotometric analysis, as described by [27]. The results were expressed in equivalent β-carotene, compared to a range of β-carotene standards prepared, starting from a stock ethanolic solution. 

Relatively to the lycopene, β-carotene, and chlorophylls, a, b determination was performed according to [28]. Essentially, 16 mL of acetone: hexane (4:6) solution was added to 1 g of fresh tomato bagasse of the two companies (C1 and C2) and shaken for 15 min. Two different phases were formed, and the hexane layer was measured at 453, 505, 645, 663 nm wavelength using a UV-vis spectrophotometer. The following equations were used to determine lycopene, β-carotene and chlorophylls a, b:

Chlorophyll a (mg/100 mL) = 0.999 × A_663_ − 0.0989 × A_645_

Chlorophyll b (mg/100 mL) = −0.328 × A_663_ + 1.77 × A_645_

Lycopene (mg/100 mL) = −0.0458 × A_663_ + 0.204 × A_645_ + 0.372 × A_505_ − 0.0806 × A_453_

β-carotene (mg/100 mL) = 0.216 × A_663_ − 1.22 × A_645_ − 0.304 × A_505_ + 0.452 × A_453_

where A_663_, A_645_, A_505,_ and A_453_ represent the absorbance at 663, 645, 505, and 453 nm each other. The results were expressed in mg/Kg DW and the determinations were performed in triplicate.

### 2.8. High-Performance Liquid Chromatography—Diode Array Detector (HPLC-DAD) Analysis 

Phenols profiles (quantitative and qualitative) of liquid fractions obtained from conventional and ohmic extraction were carried out according to [18]. Analysis was conducted on HPLC-DAD (Waters Series 600. Milford, MA, USA). A Symmetry^®^ C18 column, 250 × 4.6 mm i.d. 5 μm particle size and 125 Å pore size with a guard column (waters), was used and solvents elution consisted of solvent A – Acetonitrile (100%) with 0.2% TFA; Solvent B: acetonitrile/water (5:95 *v*/*v*) (Merck pure grade and pure water) with 0.2% TFA (Sigma-Aldrich, Germany); Samples were analysed in triplicate. Calibration curves were obtained at a detection wavelength of 280 nm to flavan-3-ols and 320 nm to flavonols. Standards solutions over the concentration range from 0.10 to 100.00 mg/L were prepared for the identification and quantification of the following compounds: rutin, naringenin, kaempferol, gallic acid, protocatechuic acid, catechin, vanillic acid, syringic acid, p-coumaric acid and phloretin (Sigma, Sintra, Portugal) expressed as µg per mL of dry weight (DW) tomato biomass. All calibration curves were linear over the concentration ranges tested, with correlations coefficients of 0.999.

Carotenoid content was also analysed by HPLC-DAD (Vydac 201TP54 C-18 column, 250 mm–4.6 mm), equipped with a C-18 pre-column. Chromatographic separation was performed as described by [29]. Solvent A with ethyl acetate (Merck pure grade) and solvent B 90:10 acetonitrile: water (Merck pure grade and pure water, 1.0 mL/min flow rate, at room temperature. The UV–vis detector was set between 270 and 550 nm. Individual carotenoids were quantified based on a calibration curve built with pure standards: β-Carotene, lycopene, and lutein (Extrasynthese, Genay Cedex, France) and expressed as milligrams per kilogram of DW.

### 2.9. LC-ESI-UHR-OqTOF-MS Analysis

An ESI source in negative mode was used to obtain the phenols profile (non-identified by HPLC) of fresh extracts according to [30]. Furthermore, UltiMate 300 Dionex UHPLC (Thermo Scientific, Waltham, MA, USA), coupled to an Ultra-High Resolution Qq-Time-Of-Flight (UHR-QqTOF) mass spectrometer with 50,000 Full-Sensitivity Resolution (FSR) (Impact II, Bruker Daltonics, Bremen, Germany). A volume of 5 µL was injected and an Acclaim RSLC 120 C18 column (100 mm × 2.1 mm, 2.2 µm) (Dionex, Sunnyvale, CA, USA) was used to identify bioactive compounds with an increased gradient elution composed of two solutions: solvent A water and 0.1% of formic acid and solvent B acetonitrile and 0.1% formic acid at a flow elution of 0.4 mL per min. The gradient started by 5% during increased to 95% in 7 min, which was maintained constant for 2 min and returned to 5% B in 1 min and maintained at 5% B for an additional 5 min at a flow rate of 0.25 mL/min. Parameters for MS analysis were set using negative ionisation mode with spectra acquired over a range from m/z 20 to 1000. The parameters were as follow: capillary voltage (4.5 kV), drying gas temperature (200 °C), drying gas flow (8.0 L/min), nebulising gas pressure (2 bar), collision RF (300 Vpp), the transfer time (120 µs) and prepulse storage (4 µs). Post-acquisition internal mass calibration used sodium formate clusters with the sodium methanoate delivered by a syringe pump at the start of each chromatographic analysis. High-resolution mass spectrometry was used to identify the phenolic compounds present in the fractions. The elemental composition for the compounds was confirmed according to accurate masse and isotope rate calculations designated as mSigma (Bruker Daltonics, Billerica, MA, USA). The accurate mass measurement was within 5 mDa of the assigned elemental composition, and mSigma values of <20 provided confirmation.

### 2.10. Statistical Analysis

All experiments were performed in triplicated and the results were expressed as the mean of triplicated analysis ± standard deviation. SPSS v. 19 (Chicago, IL, USA), was used to evaluate the statistical differences, using a non-parametric Mann-Whitney test. Furthermore, Statistica package software v. 10 (StatSoft Inc. Ibérica Lda, Oeiras, Portugal) was also applied for principal component analysis (PCA). Differences were considered significant at a 5% confidence level (*p* < 0.05). 

## 3. Results and Discussion

### 3.1. Tomato Bagasse Nutritional Analysis

#### 3.1.1. Proximal Composition

Given the aim of this study, the valorisation of tomato by-products (tomato bagasse) to obtain a suitable process with zero wastes, our work was divided into two main steps. 

Firstly, the proximal composition of tomato bagasse obtained from two processing companies was performed, as shown in Table 1. The two samples were characterized immediately after arrival from each producer (C1 and C2).

The sample provided by the companies consisted of an average of 28% seeds and 72% peels and pulp by-products. The samples were characterized in proximal composition (proteins, fat, fibre, and ash), and the results are shown in Table 1.

A significant difference was found for moisture content present in tomato bagasse from the two companies, 12.67 ± 0.24 to 67.40 ± 0.91 g/100 g DW (Refs. [7,31,32]). Different authors found quite variable results for moisture content within fresh tomato samples, some of them in the same range of the fresh sample (C1) evaluated in this study and other authors have reported a moisture content ranging from 64.3 to 92.6 g/100 g DW and 62.3 to 70.1 g/100 g DW, as reported by [7,31], respectively. Also, ref. [32] have reported even high moisture content ranging from 90.0 to 92.7 g/100 g. The variability of the reported values demonstrates the high variability associated with the moisture content found in tomato by-products, which might vary on the specific type of tomato, maturity degree, processing applied and other factors that directly affect the moisture content. This parameter may also differ if there is a previous pressure treatment before exiting the industry to reduce transport costs.

The minerals contents were similar for C1 and C2, ranging from 2.90 to 3.35 g/100 g, respectively. These values are comparable with those reported by [31] (3.92 g/100 g DW), [33] (3.3 g/100 g DW) and [7] (3.33 ± 0.02 to 4.02 ± 0.05 g/100 g dry basis) in dry tomato pomace, which one more time shows the impact of the processing applied to the samples at the final moisture content. 

Considerable amounts of protein were found in C1 compared to C2, 16.34 ± 0.62 to 19.4 ± 0.36 g/100 g DW, respectively, but the content was significantly different in samples from different companies. Other researchers also observed protein content with significant variability ranging from 10.0 to 20.9 g/100 g DW [5,7,31,34,35]. The divergences may explain the differences in companies’ processing and tomato varieties since seeds contain higher protein content than peels, and each company’s peel to seed ratio differs. 

Regarding the nutritional composition, the macronutrient present at the highest content was fibre 58.75 ± 0.42 and 57.80 ± 0.26 (g/100 g DW). Reduced differences were found among producers, and the results were comparable to the results reported by [5] 52.4 g/100 g DW. 

The differences in phytochemical profile obtained could occur for different reasons: tomato cultivars, growing conditions and the processing method (amount of seeds, pulp, and skins in the by-product), also by the differences in the processing conditions and methodologies employed by each company. As previously mentioned, the C1 was provided fresh as usually supplied for animal feeding to local farmers, and the C2 was pressed and dried under the sun to reduce the water content to considerably minimize transport costs associated with the final disposal [7,31,32]. The high fibre content in this by-product makes it a good supplement for new food formulation combined with the relatively high protein content [5].

Cell walls of tomato by-products contain pectin, a structural branched heteropolysaccharide. The total pectin present in tomato by-products was 2.11 ± 1.24 for C1 and 15.56 ± 1.41 g GUAE/100 g DW for C2. Furthermore, there are significant differences between the amount of Water-soluble Pectin (WSP) from C2 and C1 (*p* < 0.05). Other authors reported similar values of WSP, 7.0 g/100 DW of WSP extracted from tomato seeds [36] extract 8.70 ± 0.26 g/100 g of tomato paste waste. Higher values of Chelator soluble pectin (CSP) were found in C2 (*p* < 0.05) compared to C1. found Higher values of CSP (7.0 ± 1.0 g/100 g GUAE) was found by [37]. The results could be explained by the methods used to extract pectin and the tomato by-products’ conditions (C1 and C2). The WSP pectin recovered is a biopolymer weakly attached or linked by hydrogen bonds to the cell wall, which decreases the extracted yield of C1, a sample with more moisture than C2; while the pectin extracted by acid conditions is a polymer strongly attached to the cell wall through a covalent bond increasing the recovered yield compared to C2 than C1 [36]. Also, the identified differences are correlated to the tomato variety and the advanced ripening stage. Relatively to HSP, both companies present small amounts. Müller-Maatsch et al. (2016) reported values of 6.0 ± 3.0 g/100 g GUAE in [38]. The pectins were halfway esterified and partially acetylated. This pectin could be used as thickener in sauces. The differences between samples can be explained through the advanced ripening stage of tomatoes used in the processing industry and the treatment of the starting material. 

#### 3.1.2. Total Fat and Individual Fatty Acids Content

GC characterized the fatty acids composition. Both tomato by-products presented more than 10% of fatty acids, a good source of such compounds. C1 yield on the fatty acids content was 17.7%, while C2 content was 13.2%, with the most representative fraction being polyunsaturated fatty acids, with 10.1 and 8.0%, respectively (Table 2). Our values are higher than those reported in the literature. Elbadrawy and Sello found 4.0 g/100 g of the total fatty acids, while Nour et al. (2018) report 2.19 g/100 g [5,39]. The most predominant saturated fatty acid was C16:0 (palmitic acid), and the major monounsaturated fatty acid was oleic acid, and linoleic acid was the primary polyunsaturated fatty acid. Our results following the described by other authors also reported linoleic acid as the main fatty acid (52.41 g/100 g) followed by oleic acid (19.14 g/100 g) and palmitic acid (15.19 g/100 g) from tomato peel [39]. Similar results were found with tomato wastes by [5], 51.91 g/100 g of linoleic acid, followed by 18.50 g/100 g of oleic acid and 16.32 g/100 g of palmitic acid. The latter fatty acids have gained attention during the past decades due to their beneficial health effects. Besides, this by-product may be an excellent alternative to other vegetable oils, such as sunflower or soybean, due to their similar physicochemical characteristics [5,7].

### 3.2. Bioactivity Characterization

#### Antioxidant Capacity, Total Phenols Content, Total Carotenoids Content and Individual Compounds

Differences in the antioxidant activity of both tomato bagasse were found; C1 had higher antioxidant activity than C2, probably due to the initial state of the samples when receiving it at the laboratory. As described previously, one of the samples received an extra pressing and drying step, which might directly influence the antioxidant activity. The same behaviour was found when analysing the data collected from ORAC analysis, supporting the previous conclusions. Other authors found values ranging from 7.05 and 35.64 µmol TE/g DW [40]. Also, the higher tomato bagasse effect on scavenging free radicals is attributed to its higher lycopene and phenolic compounds content [5,39,41,42]. On the other hand, significant differences were found between total phenolic content from the two companies, 0.141 and 0.0924 g/100 g to C1 and C2, respectively (Table 3), which correlates well with antioxidant activity, and once again demonstrating that pressing and drying process may preserve some phenolics and consequently its relative antioxidant activity. Studies reported a positive correlation between antioxidant capacity and phenolic compounds [43]. The difference found between samples could be explained by the tomato processing steps, peeling, and dehulling, which causes degradation and, consequently, reduction of phenolic compounds. The drying process may also reduce the antioxidant compounds of tomato by-products [44]. Another critical factor is the tomato cultivar and all of the maturation processes. Similar results were found by [45], who reported an average of 0.150 g/100 g DW in two tomato by-products samples, while Nour et al. (2018) found 0.123 g GAE/100 g of DW.

No significant differences were observed between C1 and C2 samples regarding carotenoid content, which means that residual carotenoids present on bagasse are relatively resistant to the drying process. Many authors have reported the high carotenoid content in tomato processing by-products [5,34,45,46].

The high content of phenols was discriminated by HPLC, showing that the by-product samples contain differences in the phenolic compounds identified (Table 4). The flavonoids were predominant in tomato by-products from both companies.

The common phenolic compounds identified in both by-products were chlorogenic, naringenin and trans-cinnamic acids, luteolin-7-glucoside and kaempferol, the main chlorogenic acid phenolic compounds present in both extracts. Also, rutin and quercetin were found in C1 and hydroxymethylfurfural and 4-hydroxybenzoic acid in C2. The scarcity of studies with tomato by-products precludes any comparison of individual phenol compounds. Nevertheless, there is information on the effect of tomato cultivar and ripening stage on individual compounds and the compounds present during tomato processing, which helps to understand that the differences obtained in our study are also due to the cultivars used and the tomato processing [46,47].

Ellagic acid and chlorogenic acid as the most abundant phenols present in tomato by-products, 14.34 and 7.63 mg/100 g DW, respectively, was reported by [5]. The differences found in both samples depends not only on the cultivar used in the processed tomato industry but also the condition of by-products obtained (e.g., C1 and C2 samples). The results obtained are under described in the literature [48,49,50]

### 3.3. Ohmic Heating Extraction vs. Conventional Extraction

Following ohmic extraction of tomato bagasse, the samples were centrifuged to separate the liquid extract from the residue, which was then dried at 55 °C for 48 h and crushed to produce a useful flour.

#### 3.3.1. Proximate Composition

Table 5 presents the approximate composition of raw tomato samples, liquid fraction (LF) and solid fraction (SF). The solid proportion (0.80 to 0.95 g/100 g DW) presented less moisture than raw tomato bagasse (RTB). Solid fraction obtained after OH (SFOH) was greater in dried samples than solid fraction obtained after conventional method (SFCONV), with considerable differences for protein fat and fibre (*p* < 0.05).

Compared to the solid fractions, the liquid fraction had more carbohydrates (73.53 to 74.99 g/100 g DW) and ashes (10.56 to 12.45 g/100 g DW).

The increased nutritional value of SFOHs than RTB and SFCONV is due mainly to the heating process during OH treatment, which leads to the development of pores (electro-permeabilization) and intracellular diffusion of compounds which probably remained in the SF following extraction of LF.

The significant differences observed in fat and ash content of liquid fraction, extract obtained with conventional method (LFCONV) compared to SFOH probably is due to the higher affinity of compounds with chemical solvents used to their recovery.

The significantly higher protein content of SFOH than RTB and SFCONV might be associated with electric field interference, frequency, and consequently the temperature of the protein aggregation, denaturation, and soluble protein content of the matrix-induced by OH [51,52,53]. In addition, OH causes ions and other charged molecules (e.g., proteins) to move in the opposite direction of the charging electrode [51]. The impact of OH on whey protein was assessed and demonstrated that rapid joule-heating contributes to lower proteins aggregation and greater solubility of protein content in conjunction with a low electric field [54]. While, with the CONV technique, the temperature is not used, and protein denaturation does not occur. This technique also utilizes methanol with ethanol-like protein extraction results (the latter is used in OH).

SFOH also had more fatty acids than samples of RT and SFCONV (*p* < 0.05). The use of water in OH technique as a solvent is probably the principal cause for SFOH retention of higher fatty acids than SFCONV.

An excellent predictor of the nutritional value of dietary fibre is neutral detergent fibre [55]. Due to the restriction of the neutral detergent fibre technique in water-soluble fibre analysis, the fluid part was classified as “not detected”. Although this approach is restricted, the dietary fibre was another nutritionally influenced component of SF favourable for the extraction of OH. The total fibre and insoluble fibres exhibited by SFOH were greater than the SFCONV samples (*p* < 0.05).

When comparing the extraction effects of OH and CONV techniques, ref. [56] showed comparable data on corn flours. The results show that OH preserves substances (e.g., insoluble fibres), not removing the pericarp and aleurone layer during the process. In contrast, the removal of the pericarp, losses in the CONV technique, which utilizes organic solvent, may be ascribed to insoluble fibre [56]. The greater insoluble fibre content of SFOH makes OH an essential option to produce tomato flour with an enhanced bioactive profile, often linked with insoluble dietary fibre, retaining phenolic bonding fractions [56,57].

#### 3.3.2. Soluble Sugars

The total soluble sugar content of the samples of RTB and their corresponding fractions is considerably different, except for the liquid fraction (*p* < 0.05) (Table 1).

The HPLC study for soluble sugars revealed the greater content of mannose and fructose as determined by the results for tomato by-products previously shown.

The liquid fraction found that the quantity of mannose between LF and SF was considerably different. The higher concentration of mannose in LFCONV (4.10 g/100 g DW) than liquid fraction (e.g., extract obtained by OH (LFOH) and SFOH (6.5 g/100 g) can be explained by their higher fat content and a possibly higher degree of maturation of its tomatoes). At the same time, mannose rises as tomato mesocarp absorbs oil throughout the tomato ripening process.

The intended separation by fractions technique often leads to a concentration of soluble sugars, mostly mannose, in the liquid fraction. Therefore, the LF was mainly a source of minerals and carbohydrates in nutrient composition (fructose and mannose). This composition may serve as an advantage in the formulation of sports food or health-benefited food items, particularly reducing carbohydrate intake (which increases blood glucose levels) [21].

#### 3.3.3. Total Phenolic Content and Antioxidant Activity of Free and Bound Phenolics

**Figure 1:**
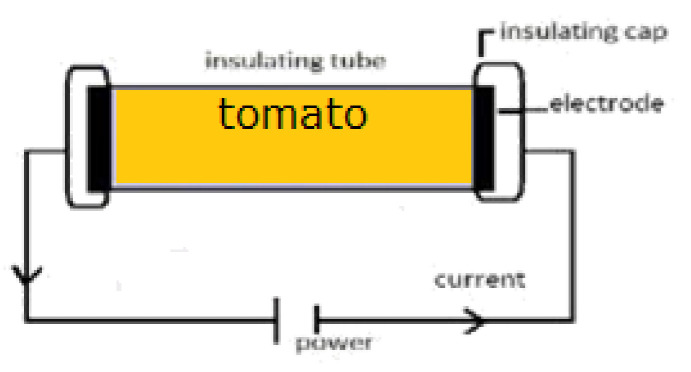
Schematic representation of OH.

Regarding total phenolic compounds, differences were found between solid fractions obtained from OH and conventional extraction methods (*p* < 0.05), see Figure 1. Nevertheless, the flour fraction presents higher total phenolic compounds than fractions submitted to an extraction process.

**Figure 2:**
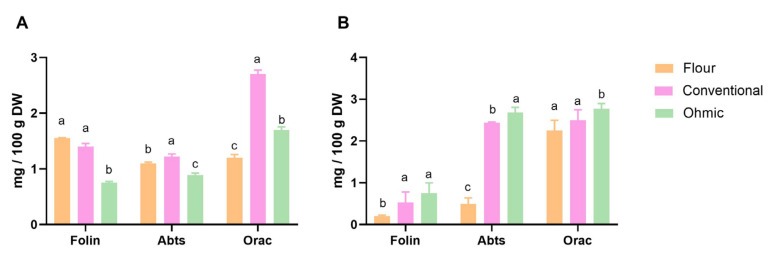
Total phenolics content, antioxidant capacity by ABTS, and ORAC method of free (**A**) and bound phenolics (**B**) from flour, conventional and OH extraction methods. The different letters in each methodology represent significant differences between samples (*p* < 0.05).

The antioxidant activity was evaluated using different methods (ABTS and ORAC), and the samples showed different behaviours (Figure 2). While in tomato flour, the free phenolics had higher antioxidant capacity, in solid factions from extracts with ohmic and conventional heating, bound phenolics showed significantly higher antioxidant activity (*p* < 0.05). This may be due to the higher overall phenolic content in these bound phenolic extracts than those at these free phenolic extracts (*p* < 0.05). Higher values of ORAC were found for different fractions; nevertheless, the same trend was found for all samples.

The significant phenols identified as bound to fibres (Table 6) from OH and conventional extracts (*p* < 0.05) were *p*-coumaric acid, gallic acid, syringic acid, vanillic acid, and catechin [58]. These phenolics acids were not extractable by aqueous methanol but issued upon alkaline hydrolysis. These results reinforce the previously presented results, whereas the bound phenolics showed significantly higher antioxidant capacity than free phenolics.

The composition of bound phenols showed an antioxidant fibre-rich in phenolic compounds resultant of flour of tomato by-products, which allows all tomato waste (peels, seeds, and pulp) to produce a product with colour, antioxidant properties, dietary fibre and fatty acids.

### 3.4. Impact of Freezing and Drying on the BC

**Figure 3:**
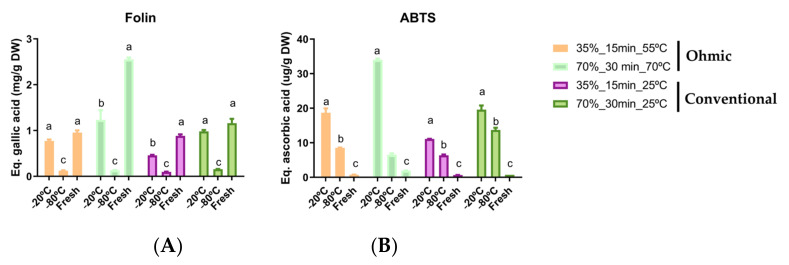
Total phenolic compounds (**A**) and total antioxidant activity (**B**) from fresh and frozen samples submitted to ohmic and conventional extractions. The different letters in each methodology represent significant differences between samples (*p* < 0.05).

The samples were frozen to understand the impact on bioactive compounds since the immediate transformation of by-products is limited to the processing conditions, and tomato processing is also seasonal. Fractions in triplicate were frozen between −20 °C and 80 °C for 24 h and compared with fresh bagasse biomass, and the results are presented in Figure 3. According to the results of total phenolics in general, the ohmic had better results than the conventional method. These results agreed with those obtained previously, where the ohmic potentiate the increase of total phenolics. Also, better results were found in fresh samples, followed by samples frozen at −20 °C.

Regarding antioxidant activity, better results were found in ohmic heating conditions with the frozen samples at −20 °C, followed by −80 °C. This result means that the frozen process improved the extraction capacity of the followed extraction processes. The freezing process generates the ice crystals within the water molecules inside of cells, and generally, the freezing through time leads to the increment of size of such crystals. The formation of crystals leads to the burst of the cellular membrane and release of inner content, but also release from the cellular membrane of phenols and bioactive molecules [59].

**Figure 4:**
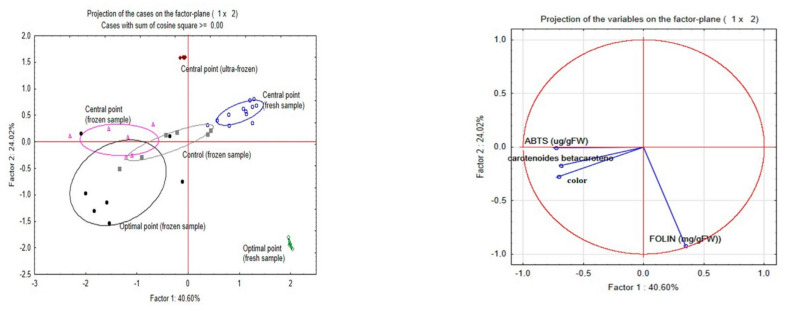
PCA analysis to principal variables, ABTS, Folin, carotenoids and colour of fresh and frozen samples.

A PCA (Figure 4) was performed to understand the impact of frozen treatments on antioxidant capacity, total phenolic compounds, and total carotenoids content. 

In the frozen samples, colour and carotenoids are correlated. The fresh samples had better results mainly in terms of phenolic compounds, and this variable has a behaviour less correlated with antioxidant activity (ABTS) and carotenoids and correlated with the behaviour above associated with the freezing process. Nevertheless, the increase in the total amount of phenolics does not directly correlate to higher antioxidant capacity and carotenoids in the extract. It is well reported that different phenolic compounds have different antioxidant activities, and the same has been reported with the different carotenoids. Also, during freezing, cell breakage can occur, leading to enzymatic reactions [16,60].

### 3.5. Prospective Valorization for Tomato Paste By-Products

Considering the research results, evaluating the amount of value-added molecules extracted and valorised would be critical to overview the tomato by-products and natural ingredients markets.

Tomato processing generates around 600 thousand to 2 million tonnes of by-products annually worldwide [7]. Considering 600 thousand tonnes as the minimum estimate of organic matter disposed of per year, and according to results present in this study, it would be possible to transform the 600 thousand tonnes of tomato by-products into 353.9 thousand tonnes of fibre, 116.9 thousand tonnes of protein, 106.2 thousand tonnes of fat, 18.0 thousand tonnes of minerals and 25.2 tonnes of carotenoids are wasted annually with higher economic costs, Table 7. If specific extraction techniques to recover phenolic compounds, carotenoids, or specific fibres, it would be possible to differentiate and increase the extracted value from tomato by-products.

Nevertheless, further studies must be performed to understand in depth the food properties and the supply value chain of these sources coming from industry in order to be able to design an efficient system to improve the performance of the application of emergent technologies, such as OH into the revalorization by-products and place food product development. It is also critical to estimate dosage levels and improve cost-benefit efficiency for a variety of applications, including food and feed additives/ingredients, cosmetics, and natural insecticides. These applications result in the acquisition of value-added goods based on the circular economy, which may help to offset the negative impacts of tomato by-products accumulating in landfills. 

The introduction of OH in the tomato processing industry’s reactor system was regarded as an aseptic process. Coupling of OH treatment with aseptic filling is a straightforward industrial solution to simplify the processing line to prepare food preserves, due to this unnecessary additional thermal management of products after business. Also, the obtained ingredients could the use of OH in the time may be specified and contributes to moving the industry away from the extractive linear structure towards a more circular economy. As a result, the acquired by-products were critical and must be valued in accordance with the principles of the circular bioeconomy.

## 4. Conclusions

One of the main goals of this research was to valorise by-products from tomato processing and characterize their main bioactive properties. All of the data collected is just the beginning of a series of experiments designed to improve an extraction process (on a food-grade basis), followed by a future application as food ingredients.

Results showed that these tomato by-products contain good phytochemical properties associated with health claims, including being rich in protein, a source of fibre, and also polyunsaturated fatty acids such as oleic acid. On the other hand, this could answer the actual trends of the sustainable economy with zero waste production. The work showed that it is possible to reduce the environmental impact of extraction processes and process costs and generate more cleaned extracts with the OH process introduced when compared to conventional extraction methods. Also, the results showed two different perspectives. With extraction methods, two fractions are obtained (a liquid and solid fraction). In the LF, free compounds (sugars and phenols mainly) were obtained, with better total phenolic compounds and antioxidant capacity than the conventional method. The OH presented better results than CONV methods regarding solid samples (obtained after the extraction process). Thus, solid fractions can also be used with high potential as flour, for example, and incorporated as ingredients in food.

Valuable by-products are disposed of annually, and this by-product is promising from a functional point of view as a good source of fibre and protein, fatty acids, carotenoids, and phenols for food formulations. Therefore, it is necessary to devise strategies for using by-products directly or obtaining different ingredients in an integral valorisation with zero waste. Thus, tomato by-products must be revalorized to improve the circular economy in this agro-industrial sector.

## Figures and Tables

**Table 1:** Proximal composition of tomato by-products.

Chemical Components		RT	
		C1	C2
Proximate composition (g/100 g)	Moisture	0.13 ± 0.24	0.67 ± 0.91 *
	Ash	2.90 ± 0.13	3.35 ± 0.01 *
	Protein	16.34 ± 0.62	19.41 ± 0.36 *
	Fat	16.52 ± 0.35 *	13.20 ± 0.50
	Crude Fiber	58.75 ± 0.42	57.80 ± 0.26
	Total Sugars	5.76 ± 0.28	5.18 ± 0.16
Fiber	NDF	30.01 ± 0.03 *	16.93 ± 0.04
	ADF	12.98 ± 0.64 *	8.60 ± 0.23
Total Lignin		9.80 ± 0.23	12.73 ± 0.49 *
Cellulose (as glucose)		11.27 ± 0.66 *	7.23 ± 0.25
Hemicellulose		19.58 ± 0.10 *	9.08 ± 0.13
Pectins	Water soluble Pectin (WSP)	6.10 ± 0.81	11.68 ± 0.73 *
	Chelator soluble pectin (CSP)	5.04 ± 0.39 *	2.87 ± 0.40
	Hydroxide Soluble Pectin (HSP)	0.97 ± 0.04	1.01 ± 0.28

The values are expressed at g/100 g DW (dry weight); C1 and C2 represent the two companies studied. * *p* < 0.05 significant differences between the same parameters among company’s samples (C1 and C2 composition).

**Table 2:** Fatty acids profile (identification and quantification) of tomato by-products.

Fatty Acid Name	C1	C2
Lauric acid (C12:0)	0.01	n.d.
Myristic acid (C14:0)	0.04	n.d.
Myristoleic acid (C14:1)	n.d.	n.d.
Pentadecanoic acid (C15:0)	0.01	n.d.
Cis-10-Pentadecanoic acid (C15:1)	0.01	n.d.
Palmitic acid (C16:0)	2.50	1.95
Palmitoleic acid (C16:1)	0.05	0.03
Heptadecanoic acid (C17:0)	0.02	0.01
Cis-10-Heptedecenoic acid (C17:1)	0.01	0.06
Stearic acid (C18:0)	1.03 ^X^	0.67
Elaidic acid (C18:1n9t)	n.d.	2.41 ^X^
Oleic acid (C18:1n9c)	3.82 ^X^	n.d.
Linoleic acid (C18:2n6c)	9.64 ^X^	7.62
Arachidic acid (C20:0)	0.09	0.05
y-Linoleic acid (C18:3n6)	0.43	0.35
Cis-11Eicosenoic acid (C20:1)	0.02	0.01
Heneicosanoic acid (C21:0)	0.01	n.d.
cis-11,14-Eicosadienoic acid (C20:2)	0.02	n.d.
Behenic acid (C22:0)	n.d.	0.01
Lignoceric acid (C24:0)	0.03	0.02
Total (mg FA/100 g DW)	17.71	13.20
Total saturated (mg FA/100 g DW)	3.78	2.72
Total monounsaturated (mg FA/100 g DW)	3.89	2.51
Total polyunsaturated (mg FA/100 g DW)	10.07	7.97

c/t, cis/trans double bond; DHA, docosahexaenoic acid; n.d.: not detected. <LOQ, concentration under quantitation limit. ^X^ Superscript letter in a value for significant differences in a same lipid fraction among raw C1 and C2 composition (*p* < 0.05). The values are expressed at g FA/100 g DW (dry weight); C1 and C2 represent the two companies studied.

**Table 3:** Antioxidant capacity and total phenolic content.

Sample	C1	C2
Total Phenolic compounds (GAE) *	0.14 ± 0.06 ^X^	0.092 ± 0.0145
Total carotenoids content (β-carotene Equivalent) *	0.780 ± 0.12	0.67 ± 0.0073
ABTS (AAE) *	0.115 ± 0.016	0.858 ± 0.024 ^X^
ORAC (trolox eq.) **	2.31 ± 0.020	2.09 ± 0.011

* The values are expressed as g/100 g DW (dry weight); ** expressed at µmol/TE 100 g C1 and C2 represent the two companies studied. ^X^ Superscript letter in a value for significant differences in a same fraction among raw C1 and C2 composition (*p* < 0.05).

**Table 4:** Individual compounds identified by HPLC from the two companies.

Samples	Compounds Identified	Concentration (mg/100 g DW)
C1	Rutin	1.54 ± 0.01
	Quercetin	0.14 ± 0.01
	Naringenin acid	0.0086 ± 0.01
	Transcinnamic acid	0.12 ± 0.01
	Chlorogenic acid	4.12 ± 0.02
	Luteolin-7-glucoside	n.q.
	Kaempferol	n.q.
C2	4-hydroxybenzoic acid	0.11 ± 0.02
	Naringenin acid	0.00178 ± 0.01
	Trans-cinnamic acid	0.12 ± 0.01
	Chlorogenic acid	2.72 ± 0.01
	Luteolin-7-glucoside	n.q.
	Kaempferol	n.q.

n.q. non-quantified.

**Table 5:** Chemical composition of raw tomato bagasse (RTB) and tomato fractions (LF-T and SF-T) obtained after OH and CONV extractions (g/100 g DW).

		RTB	LF-T		SF-T	
Chemical Components			LFOH	LFCONV	SFOH	SFCONV
Proximate composition (g/100 g)	Moisture	0.95 ± 0.07	3.08 ± 0.03	3.14 ± 0.07	0.80 ± 0.09	0.90 ± 0.08
	Ash	3.16 ± 0.10	10.56 ± 1.22	12.45 ± 0.22	3.02 ± 0.12	2.98 ± 0.16
	Protein	17.65 ± 0.30	4.01 ± 0.15	4.32 ± 0.23	17.44 ± 0.25 *	16.29 ± 0.59
	Fat	15.49 ± 0.90	4.22 ± 1.25	6.46 ± 0.87	19.02 ± 0.43 *	17.82 ± 0.30
	Crude FiberFiber	57.60 ± 1.54	0.15 ± 0.08	0.10 ± 0.06	60.47 ± 0.44 *	59.06 ± 0.67
	Carbohydrates	5.70 ± 1.23	74.99 ± 2.23	73.53 ± 2.19	1.22 ± 0.34	2.91 ± 0.98
FiberFiber	IDF	24.42 ± 0.96	n.d	n.d	48.06 ± 0.11	46.01 ± 0.13
	SDF	11.13 ± 0.75 ^b^	10.86 ± 0.85 ^b^	12.98 ± 0.64 ^a^	n.d	n.d
Klason Lignin		11.34 ± 1.49	*	*	13.06 ± 0.52 ^b^	14.09 ± 0.27 ^a^
Structural Carbohydrates	Cellulose (as glucose)	9.14 ± 1.66 ^b^	*	*	13.74 ± 1.12 ^a^	12.82 ± 0.87 ^a^
	Hemicellulose	14.29 ± 0.12 ^b^	*	*	25.2 ± 0.10 ^a^	24.72 ± 0.30 ^a^
	Xylose	14.69 ±	*	*	13.2 ± 0.10 ^a^	8.89
	Galactose	0.75 ± 0.08	*	*	0.12 ± 0.02	0.31 ± 0.021
	Mannose	6.64 ± 0.05	*	4.10 ± 0.12 ^a^	6.5 ± 0.18	*
	Fructose	3.25 ± 1.23	2.24 ± 0.87	0.99 ± 0.12	0.61 ± 0.05	2.74 ± 0.22
Resistant Protein		17.04 ± 0.09	n.d	n.d	16.03 ± 0.05 ^a^	11.69 ± 0.03 ^b^

R-T—raw tomato by-products; LF-T—liquid fraction from tomato bagasse; SF-T—solid fraction from tomato bagasse; LFOH—liquid fraction obtained in the OH extraction; LFCONV—liquid fraction obtained after CONV method; SFOH—solid fraction obtained after OH; SFCONV—solid fraction obtained after CONV method; IDF—insoluble dietary fibre; SDF—soluble dietary fibre. n.d.:Not detected. * 1 g Glucose equivalent/100 g sample dry weight. Data were expressed as mean ± SD (*n* = 3). The different superscripts in the same row represent significant differences between samples (*p* < 0.05).

**Table 6:** Bound phenols complexed with tomato bagasse fibre, comparative analysis between different extraction techniques. Qualitative and quantitative analysis by HPLC.

Phenolic Compounds	CONV	OH
4-hydroxybenzoic acid	2.14 ± 0.01	2.41 ± 0.08
Caffeic acid	3.36 ± 0.51	n.d.
Vanillin	2.64 ± 0.02	1.14 ± 0.01
p-coumaric acid	4.04 ± 0.02	2.14 ± 0.32
Rutin	8.04 ± 0.71	0.58 ± 0.001

The values are expressed at g/100 g DW (dry weight); CONV – Conventional extraction; OH—Ohmic heating extraction; n.d.—non-detected.

**Table 7:** Amount of valuable ingredients can be recovered in 600 thousand tomato processing by-products.

Proximal Composition	(g/1 Kg)	Content (Thousand Tonnes)
Fiber	590	353.9
Protein	195	116.9
Fat	178	106.9
Minerals	30	18.0
Carotenoids	4	0.025

## Data Availability

Data is contained within the article.
